# Glyoxalase activity in human erythrocytes and mouse lymphoma, liver and brain probed with hyperpolarized ^13^C-methylglyoxal

**DOI:** 10.1038/s42003-018-0241-1

**Published:** 2018-12-21

**Authors:** Dmitry Shishmarev, Philip W. Kuchel, Guilhem Pagès, Alan J. Wright, Richard L. Hesketh, Felix Kreis, Kevin M. Brindle

**Affiliations:** 1 The Australian National University, John Curtin School of Medical Research, Canberra, ACT Australia; 2 The University of Sydney, School of Life and Environmental Sciences, Sydney, NSW Australia; 3 INRA, AgroResonance – UR370 Qualité des Produits Animaux, F-63122, Saint Genès Champanelle France; 4 University of Cambridge, Cancer Research UK Cambridge Institute, Li Ka Shing Centre, Cambridge, UK

**Keywords:** Metabolomics, Cancer metabolism, Diagnostic markers, Enzymes

## Abstract

Methylglyoxal is a faulty metabolite. It is a ubiquitous by-product of glucose and amino acid metabolism that spontaneously reacts with proximal amino groups in proteins and nucleic acids, leading to impairment of their function. The glyoxalase pathway evolved early in phylogeny to bring about rapid catabolism of methylglyoxal, and an understanding of the role of methylglyoxal and the glyoxalases in many diseases is beginning to emerge. Metabolic processing of methylglyoxal is very rapid in vivo and thus notoriously difficult to detect and quantify. Here we show that ^13^C nuclei in labeled methylglyoxal can be hyperpolarized using dynamic nuclear polarization, providing ^13^C nuclear magnetic resonance signal enhancements in the solution state close to 5,000-fold. We demonstrate the applications of this probe of metabolism for kinetic characterization of the glyoxalase system in isolated cells as well as mouse brain, liver and lymphoma in vivo.

## Abstract (web summary)

Shishmarev et al. use rapid dissolution dynamic nuclear polarization to monitor glyoxalase activity in a non-invasive manner using nuclear magnetic resonance spectroscopy. They apply their method to monitor and quantify metabolism of methylglyoxal in human red blood cells and mouse tissues.

## Introduction

Methylglyoxal (MeGx) is formed both inside cells^1–3^ and in blood plasma^4–6^, primarily as a by-product of glycolysis^7,8^ and amino acid metabolism^9^, with a total daily production estimated at 0.2–1.2 g in the adult human^8,10^. As a ketoaldehyde, MeGx is highly reactive and spontaneously modifies side chain amino groups in proteins and nucleic acids, leading to impairment of their function and degradation^11^. Initial catabolism of MeGx occurs via the glyoxalase pathway^12,13^, which consists of glyoxalase I (Glo1, enzyme commission number 4.4.1.5, lactoylglutathione lyase) and glyoxalase II (Glo2, enzyme commission number 3.1.2.6, hydroxyacylglutathione hydrolase), which in red blood cells (RBCs) have a maximum flux capacity ~100 times that of glycolysis^14,15^. The pathway requires reduced glutathione (GSH) as a co-substrate and converts MeGx into D-lactate (D-Lac) (as opposed to the L-lactate produced by glycolysis in higher organisms^16^), which is then further metabolized in peroxisomes^17^. Understanding the fate of MeGx in biology and its role in disease development is beginning to emerge^18^. Additional functions of MeGx and the glyoxalase pathway in cell physiology remain largely unresolved.

The kinetics of MeGx catabolism has been studied previously in dilute lysates of RBCs using ^1^H nuclear magnetic resonance (NMR) spectroscopy^15^. However, due to poor intrinsic sensitivity of NMR and the need to accumulate each NMR spectrum for ~4 min, the glyoxalase reactions appeared to be too fast to study in intact cells and tissues (going to completion in <1 min). We demonstrate here, using rapid dissolution dynamic nuclear polarization (RD-DNP)^19^, that it is possible to monitor the glyoxalase pathway non-invasively in whole cells and tissues, on the sub-minute timescale. Using hyperpolarization, we achieve ~5,000-fold enhancements in NMR sensitivity, which allows detection and quantification of the glyoxalase reactions in RBCs and mouse tissues in vivo.

## Results

### Synthesis of ^13^C-labeled methylglyoxal

**Fig. 1:**
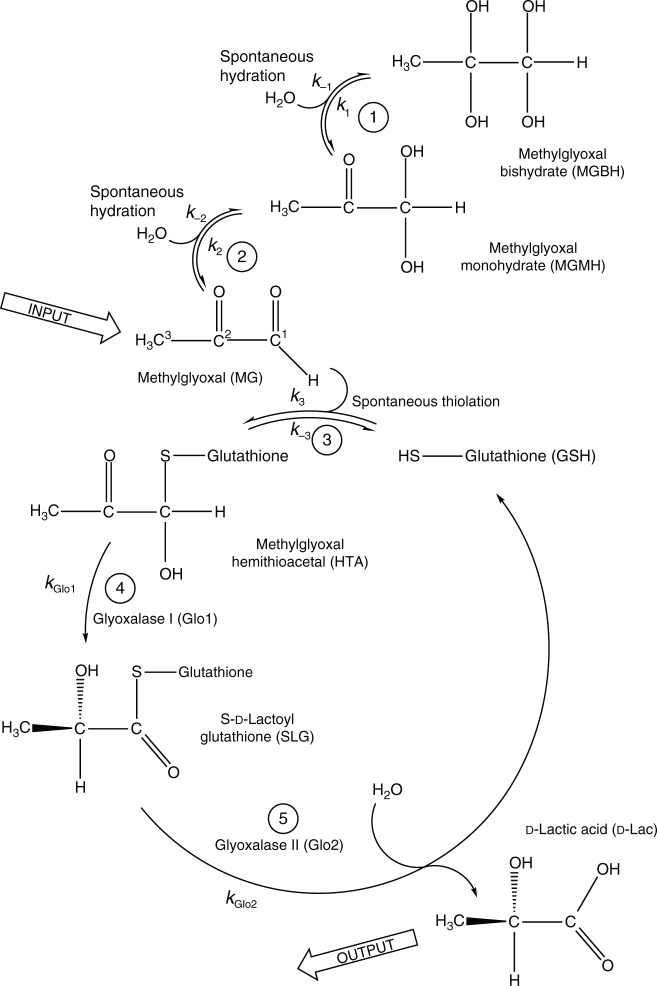
Glyoxalase pathway of methylglyoxal catabolism. Ketoaldehyde form of methylglyoxal (MG) is spontaneously thiolated by reduced glutathione (GSH) (reaction 3), which is followed by the two reactions catalyzed by glyoxalase I (4) and glyoxalase II (5), producing S-D-lactoylglutathione (SLG) and D-lactic acid (D-Lac), respectively. There are also two spontaneous hydration reactions of the keto-aldehyde form, (1) and (2)

Two isotopomers of ^13^C-labeled MeGx, [2-^13^C]MeGx and [1,3-^13^C]MeGx, were synthesized from [2-^13^C]acetone and [1,3-^13^C]acetone, respectively. The synthetic procedure was adapted from a method for radiolabeling MeGx^20^ and described in Methods. Since the ^13^C nucleus at the C2 position in MeGx does not have any attached protons (Fig. 1), it has a much larger NMR longitudinal relaxation time *T*_1_ and hence longer nuclear polarization lifetime than the C1 or C3 carbons. Consequently, we observed better spectral quality in RD-DNP time courses obtained with hyperpolarized [2-^13^C]MeGx and therefore focused on this isotopomer. (Results obtained with [1,3-^13^C]MeGx are shown in Supplementary Figure 1.) The purity of the synthesized [2-^13^C]MeGx was confirmed by one-dimensional (1D) ^1^H (Supplementary Figure 2), 1D ^13^C (Supplementary Figure 3) and two-dimensional (2D) ^1^H-^13^C HMBC (Supplementary Figure 4) NMR spectra. MeGx is spontaneously hydrated (Fig. 1) and ^1^H and ^13^C NMR spectra confirmed that it exists as three interchanging (on the sub-minute timescale) forms in aqueous solution, viz., free keto-aldehyde (MG), the monohydrate (MGMH), and the bishydrate (MGBH), which at 37 ^o^C were in the ratio 1:71:28 (keto-aldehyde:MGMH:MGBH).

### Detection of methylglyoxal catabolism in whole cells

**Fig. 2:**
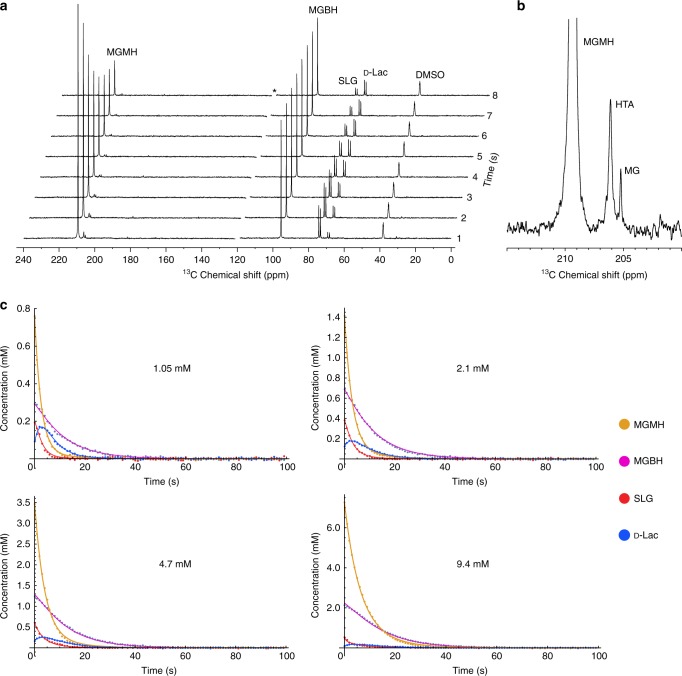
RD-DNP time courses acquired following injection of hyperpolarized [2-^13^C]methylglyoxal into a suspension of RBCs. **a** ^13^C NMR spectra obtained upon injection of 1 mL of hyperpolarized [2-^13^C]MeGx into 3 mL of RBCs (hematocrit, *Ht*, of 52%) in phosphate-buffered saline giving a final concentration of 1.3 mM. Spectra were acquired every 1 s. *Denotes position of spectral artifact that was edited out. **b** Expansion of the 200-215 ppm region of the bottom spectrum in shown in **a**. **c** ^13^C NMR peak intensities of the four main species in spectra acquired following injection of different concentrations of [2-^13^C]MeGx into suspensions of RBCs (final *Ht* values 39–40%). Data points were scaled so that the initial sums of the peak amplitudes of MGMH and MGBH were equal to the nominal concentrations of injected MeGx. The solid lines are nonlinear least squares fits of numerical solutions of the array of differential equations. Abbreviations: D-Lac, [2-^13^C]D-lactate; HTA, hemithioacetal of [2-^13^C]MeGx; MG, the ketoaldehyde form of [2-^13^C]MeGx; MGBH, the bishydrate of [2-^13^C]MeGx; MGMH, the monohydrate of [2-^13^C]MeGx; SLG, [2-^13^C]S-D-lactoylglutathione; DMSO, natural abundance ^13^CD_3_ in the dimethylsulfoxide-d6 used to make up the hyperpolarization solution

A solution of hyperpolarized [2-^13^C]MeGx was injected into an RBC suspension that was thermally pre-equilibrated at 37 °C inside the NMR spectrometer bore, and a series of 1D ^13^C NMR spectra (1 s per spectrum) was acquired using a small flip angle (~4°) excitation pulse. The emergence of SLG and D-Lac, the respective products of Glo1 and Glo2, demonstrated rapid catabolism of MeGx by the glyoxalase pathway in RBCs (Fig. 2a).

Hyperpolarization led to high spectral signal-to-noise ratios for all the MeGx species (~200:1 for MGMH), despite the [2-^13^C]MeGx concentration in the sample of only 1.3 mM. In comparison, a signal-to-noise ratio of ~12:1 was achieved for MGMH in ^13^C-NMR spectra of non-hyperpolarized ~40 mM [2-^13^C]MeGx in D_2_O, recorded from 64 transients in 4 min (Supplementary Figure 3). Thus, the DNP signal enhancement was ~5000.

The MGMH resonance declined rapidly due to glyoxalase-mediated flux and longitudinal relaxation, whereas the MGBH resonance declined more slowly despite a similar *T*_1_ (see below for estimates of these parameter values), primarily because MGMH is the intermediate that feeds rapidly onto the free ketoaldehyde (MG) and the rest of the pathway, while exchange between the two hydrated species is relatively slower. The smaller resonances from the ketoaldehyde and hemithioacetal (HTA) persisted in the spectra (Fig. 2b), consistent with continuing regeneration of these minor forms during the flow of ^13^C label from MGMH/MGBH via MG/HTA to SLG and D-Lac (Fig. 1). SLG was prominent initially (at ~2 s) and declined thereafter, consistent with a very fast build-up catalyzed by Glo1, followed by removal in the reaction catalyzed by Glo2, with concurrent loss of signal due to *T*_1_ relaxation.

The emergence of D-Lac occurred more slowly than for SLG, as expected for a metabolite that is further downstream. A similar pattern of labeling was evident in RBC lysates (Supplementary Figure 5) and in suspensions of murine lymphoma EL4 cells (Supplementary Figure 6).

### Quantification of glyoxalase kinetics in whole RBCs

Figure 2c shows the temporal evolution of ^13^C-labeled MGMH, MGBH, SLG and D-Lac, obtained upon injection of four different amounts of [2-^13^C]MeGx (final concentrations of 1.05, 2.1, 4.7 and 9.4 mM). The time at which the maximum D-Lac peak intensity occurred varied only slightly with changes in the concentration of [2-^13^C]MeGx. The extent of reaction, indicated by the relative maximum amplitude of the D-Lac signal, was highest for the lowest starting concentration of MeGx. Also, at higher substrate concentrations the glyoxalase system became partially saturated. The MGMH signal showed an apparent single-exponential decay, as expected for the main substrate species, while MGBH showed the appearance of a bi-exponential decay, consistent with ^13^C label flowing through MGMH prior to yielding the other two products. SLG was formed so fast that its maximum amplitude occurred shortly after MeGx addition; while D-Lac showed the rise-and-fall profile expected for a product at the end of a reaction sequence, as has previously been observed^21–24^.

The rate constants describing ^13^C label flux were estimated by iterative fitting of the following (continuous) differential equations, which were integrated numerically, to the signal intensities in Fig. 2c:

$${\hskip-0.5pt}\frac{{{\rm{d}}[{\mathrm{MGBH}}^ \ast ]}}{{{\rm{d}}t}} = - \frac{1}{{T_1^{{\mathrm{MGBH}}}}}\left[ {{\mathrm{MGBH}}^ \ast } \right] \! - \! k_1\left[ {{\mathrm{MGBH}}^ \ast } \right] + k_{ - 1}\left[ {{\mathrm{MGMH}}^ \ast } \right]$$

$${\hskip-4pc}\frac{{{\rm{d}}[{\mathrm{MGMH}}^ \ast ]}}{{{\rm{d}}t}} = - \frac{1}{{T_1^{{\mathrm{MGMH}}}}}\left[ {{\mathrm{MGMH}}^ \ast } \right] - k_{{\mathrm{Glo}}1}\left[ {{\mathrm{MGMH}}^ \ast } \right] \\ - k_{ - 1}\left[ {{\mathrm{MGMH}}^ \ast } \right] + k_1\left[ {{\mathrm{MGBH}}^ \ast } \right]$$

$$\frac{{{\rm{d}}\left[ {{\mathrm{SLG}}^ \ast } \right]}}{{{\rm{d}}t}} = - \frac{1}{{T_1^{{\mathrm{SLG}}}}}\left[ {{\mathrm{SLG}}^ \ast } \right] - k_{{\mathrm{Glo}}2}\left[ {{\mathrm{SLG}}^ \ast } \right] + k_{{\mathrm{Glo}}1}\left[ {{\mathrm{MGMH}}^ \ast } \right]$$

$${\hskip-6.1pc}\frac{{{\rm{d}}[{\mathrm{Lac}}^ \ast ]}}{{{\rm{d}}t}} = - \frac{1}{{T_1^{{\mathrm{Lac}}}}}\left[ {{\mathrm{Lac}}^ \ast } \right] + k_{{\mathrm{Glo}}2}[{\mathrm{SLG}}^ \ast ],$$

where *T*_1_^MGBH^, *T*_1_^MGMH^, *T*_1_^SLG^, *T*_1_^Lac^ are the longitudinal relaxation times of the indicated species; *k*_1_, *k*_−1_, *k*_Glo1_, *k*_Glo2_, are the kinetic rate constants shown in Fig. 1 and [MGBH*], [MGMH*], [SLG*], and [Lac*] are the concentrations of the corresponding hyperpolarized species. The solid lines in Fig. 2c show the fitting results.

Initially, we considered RD-DNP time courses from samples in which cell suspensions had low cytocrits (3–9.5%). Due to the low overall glyoxalase activity, the ^13^C-NMR signal intensities in such spectral series were dominated by just two species, MGBH and MGMH (e.g., Supplementary Figure 6b), which allowed independent estimation of the hydration-exchange constants *k*_1_ and *k*_−1_ as well as the two *T*_1_ values: *k*_1_ = 0.039 ± 0.009 s^−1^; *k*_−1_ = 0.015 ± 0.005 s^−1^; *T*_1_^MGBH^ = 19.3 ± 5.3 s and *T*_1_^MGMH^ = 16.0 ± 1.9 s. The obtained best-fit parameters for each trial are given in Supplementary Tables 1-8. The estimated values for *k*_1_ and *k*_−1_ compare favorably with those reported previously for dilute hemolysates, 0.051 ± 0.004 s^−1^ and 0.021 ± 0.004 s^−1^, estimated using a magnetization transfer method^15^.

The estimated values of *T*_1_^MGBH^, *T*_1_^MGMH^, *k*_1_ and *k*_−1_ were then fixed in subsequent regression trials in which the other intermediate metabolites had much higher concentrations due to higher cytocrit values (39–40%). Fitting of the kinetic model to the data shown in Fig. 2c gave a *T*_1_^SLG^ of 0.9 ± 0.3 s, which was consistent with the presence of a proton directly bonded to ^13^C in the lactoyl moiety, which provides an efficient dipole-dipole relaxation pathway. The *T*_1_ of [2-^13^C]D-lactate was also small compared with those of the methylglyoxal species, at 5.3 ± 1.2 s.

**Fig. 3:**
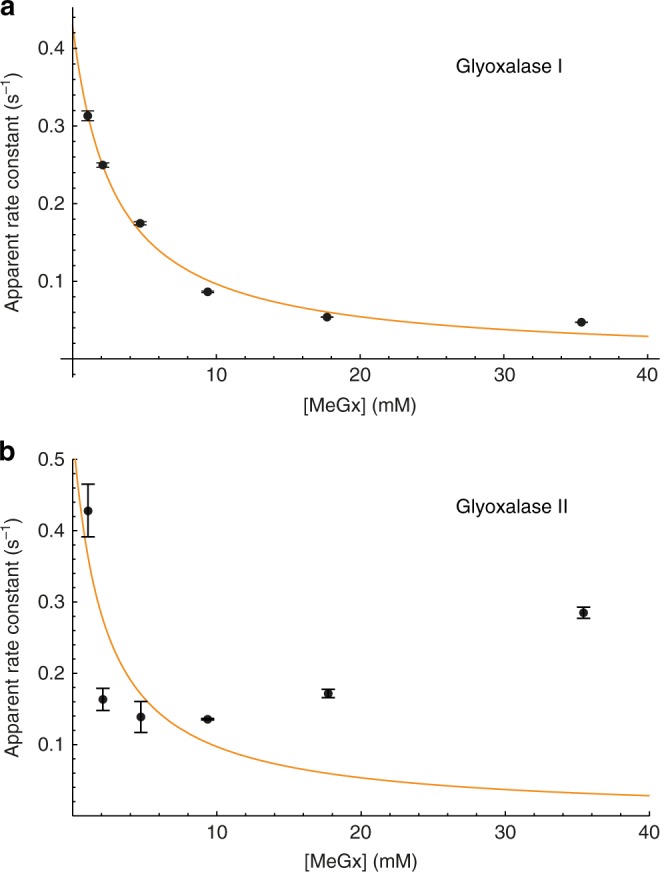
Apparent rate constants of the two glyoxalase reactions in RBC suspensions as functions of total concentration of injected methylglyoxal. The data were derived from fitting the time courses of ^13^C NMR peak intensities shown in Fig. 2c, plus two data sets obtained with [MeGx] = 17.5 and 34.5 mM (Supplementary Figure 7). **a** Black discs represent the best-fit values of *k*_GLO1_, given in Supplementary Table 9 (mean ± SE). The solid orange line is a nonlinear least squares regression of the Michaelis–Menten expression for glyoxalase 1, giving $V_{{\mathrm{max}},{\mathrm{app}}}^{{\mathrm{Glo}}1}$ of 1.2 ± 0.4 mM s^−1^ and a *K*_m,app_ of 2.9 ± 1.3 mM. **b** Black discs represent the best-fit values of *k*_GLO2_, given in Supplementary Table 9 (mean ± SE). The solid orange line is an attempt at nonlinear least squares regression of the Michaelis–Menten expression for glyoxalase 2

Estimates of the glyoxalase (*k*_Glo1_ and *k*_Glo2_) rate constants were obtained by fitting the data shown in Fig. 2c, plus two additional data sets with the initial total MeGx concentration of 17.7 and 35.4 mM (Supplementary Figure 7). Best-fit parameters obtained by fitting the Bloch-McConnell model to these data are summarized in Supplementary Table 9. Figure 3a shows the dependence of the apparent rate constant for the reaction catalyzed by Glo1 (*k*_Glo1_) on the total initial concentration of MeGx. Regressing the Michaelis–Menten equation onto the data of Fig. 3a yielded an apparent $V_{{\mathrm{max}},{\mathrm{app}}}^{{\mathrm{Glo}}1}$ of 1.2 ± 0.4 mM s^−1^ and *K*_m,app_ of 2.9 ± 1.3 mM. Since HTA is the true substrate for Glo1, the enzyme kinetic parameters were converted according to formulae derived in Methods: $V_{{\mathrm{max}}}^{{\mathrm{Glo}}1} = \frac{{V_{{\mathrm{max}}}^{{\mathrm{Glo}}1,{\mathrm{app}}}[{\mathrm{GSH}}]}}{{[\left( {K_1 + 1} \right)K_2 + 1]K_3}}$, which gave (assuming [GSH] = 2.2 mM in the RBCs^15,25^) $V_{{\mathrm{max}}}^{{\mathrm{Glo}}1}$ of 0.22 ± 0.07 mM s^−1^, or 33.4 ± 10.6 mmol min^−1^ (L RBC)^−1^. Similarly, the apparent *K*_m_ for MGMH was converted to that for HTA via the expression derived in Methods: $K_{\mathrm{m}}^{{\mathrm{Glo}}1} = \frac{{K_{\mathrm{m}}^{{\mathrm{Glo}}1,{\mathrm{app}}}}}{{\frac{{K_{\mathrm{m}}^{{\mathrm{Glo}}1}[\left( {K_1 + 1} \right)K_2 + 1]K_3}}{{[{\mathrm{GSH}}]}}\left( {1 + \frac{{[{\mathrm{GSH}}]}}{{K_{\mathrm{i}}^{{\mathrm{GSH}}}}}} \right)}}$, which gave $K_{\mathrm{m}}^{{\mathrm{Glo}}1}$= 0.42 ± 0.19 mM. The latter value is very close to the previously reported *K*_m_ for Glo1, 0.46 mM^15^, while $V_{{\mathrm{max}}}^{{\mathrm{Glo}}1}$ is smaller by a factor of two than in dilute hemolysates^15^. This discrepancy can be partly explained by a large accumulation of non-hyperpolarized, and hence invisible, methylglyoxal thus leading to an underestimate of the true carbon flux.

The enzyme kinetic plot for Glo2 (Fig. 3b) displayed less convincing Michaelis–Menten dependence of the apparent rate constant on the (total MeGx) substrate concentration. The scatter and standard errors of the experimental data were large, leading to unreliable parameter estimates, viz., $V_{{\mathrm{max}}}^{{\mathrm{Glo}}2,{\mathrm{app}}}$ = 1.2 ± 2.2 mM s^−1^ and $K_{\mathrm{m}}^{{\mathrm{Glo}}2,{\mathrm{app}}}$ = 2.2 ± 7.3 mM.

### Detection of glyoxalase activity in vivo

**Fig. 4:**
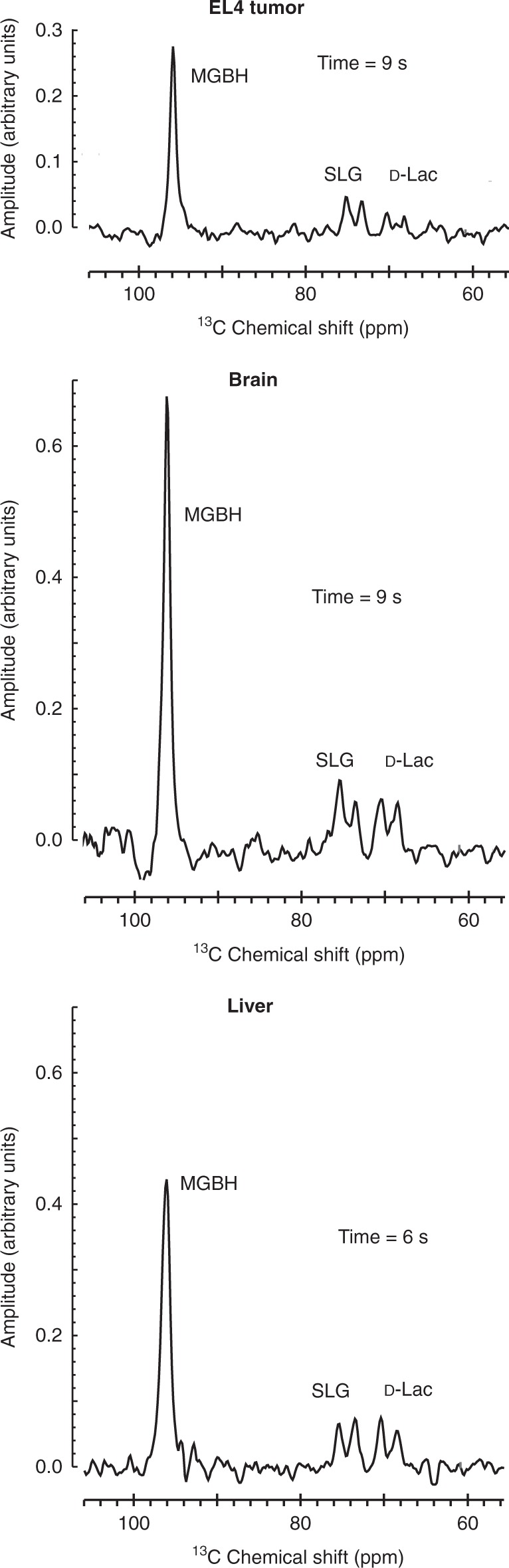
^13^C NMR spectra acquired from the liver, brain, and an EL4 tumor of three different mice following injection of hyperpolarized [2-^13^C]MeGx. Spectra were acquired every 1 s for 180 s. The spectra shown are those that had the maximum resonance intensity for SLG and D-Lac. Full time-courses are given in Supplementary Figures 8–10

Following experiments on suspensions of murine lymphoma EL4 cells, where we observed reaction time courses similar to those in RBCs (Supplementary Figure 6), we studied [2-^13^C]MeGx metabolism in EL4 tumors implanted subcutaneously in mice (*n* = 3), as well as mouse liver and brain. A solution of hyperpolarized [2-^13^C]MeGx was injected into the tail vein of mice that were positioned in a horizontal bore 7 T magnet; and NMR signals were detected using a 2 cm diameter surface coil placed over the tissue of interest. As in the cell suspension studies, rapid appearance of the glyoxalase products was detected in all studied tissues. Spectra that had maximum intensities for SLG and D-Lac are shown in Fig. 4 (all spectra acquired for up to 20 s are shown in Supplementary Figures 8-10).

The ^13^C NMR spectra from EL4 tumors (Supplementary Figure 8) were similar to those from brain (Supplementary Figure 9) and liver (Supplementary Figure 10), but a notable feature was the slower rise of the ^13^C signals, with MGBH only appearing after 4 s in contrast to 2 s in the other two tissues. D-Lac appeared before other spectral features at 3 s and was still present after 18 s. SLG_max_ was observed at 9 s, later than in both the liver and the brain. Overall, the EL4 tumors appeared to metabolize [2-^13^C]MeGx at a rate comparable to liver and brain, but after a further 2 s delay, that is consistent with the longer vascular path from the heart to the dermis on the flank of the animal, and then into the tumor. The rates of D-Lac production in the three tissues were similar to those observed in RBC suspensions.

## Discussion

Production of d-Lac in the glyoxalase pathway involves a sequence of *four* reactions (dehydration of hydrated MeGx → thiolation of the ketoaldehyde by GSH → elimination/lyation via Glo1 → hydrolysis via Glo2), which is amongst the longest sequences of reactions observed using a hyperpolarized ^13^C-labeled substrate. Another example of a system involving four sequential reactions is detection of ^13^C label from [2-^13^C]pyruvate in citrate and glutamate, having passed via acetyl-CoA and isocitrate, respectively;^26^ and hyperpolarized [U-^13^C]d-glucose produces detectable amounts of dihydroxyacetone phosphate and l-lactate via the 11 reactions of glycolysis^27^. Because the metabolism of MeGx lies outside the mainstream energy-transducing pathways, it is like another renegade or faulty metabolite, 2-hydroxyglutarate, excessive accumulation of which is implicated in cancer^28^. These types of metabolites are appealing as probes of altered gene expression, capable of distinguishing one tissue from another, and neoplastic cells from their normal counterparts. Glyoxalase I is known to be overexpressed in tumors^29^ and has been proposed as a prognosis factor for cancer progression^30^. ^13^C-labeled d-lactate could also be hyperpolarized and used to track its fate in various tissues using similar methodology (or with local catheterization and perfusion of an organ or tumor). [2-^13^C]MeGx could also be used as a precursor of endogenously produced [2-^13^C]d-lactate for this purpose.

Overall, we demonstrated that kinetic probing of the glyoxalase pathway is possible by using the RD-DNP method, with potential clinical imaging applications. We developed a reliable method for the synthesis of [2-^13^C]MeGx and demonstrated hyperpolarization of this material using RD-DNP methodology, with signal enhancements of ~5000-fold. We showed that hyperpolarized [2-^13^C]MeGx can be used to probe glyoxalase activity in RBCs, murine lymphoma EL4 cells in vitro, as well as mouse brain, liver and EL4 tumors in vivo. The glyoxalase kinetic parameters obtained in RBCs showed good agreement with those obtained previously using other NMR and light-spectroscopy methods, although the estimated maximal velocity of Glo1 was 2-fold lower than expected, a fact that requires further investigation. Finally, [2-^13^C]MeGx, combined with RD-DNP and ^13^C NMR, holds promise as an in vivo probe of differential glyoxalase expression, and hence the basis of discriminating between normal and pathological tissues.

## Methods

### Chemicals and solutions

Analytical reagents were from Sigma-Aldrich (St Louis, MO, USA) unless stated otherwise. [2-^13^C]Acetone and [1,3-^13^C]acetone were from Cambridge Isotope Laboratories (Andover, MA, USA). Osmolalities of cell media were adjusted to 290 ± 5 mOsm kg^−1^ using a vapor pressure osmometer (Model 5520, Wescor Instruments, Logan, UT, USA).

### Synthesis of ^13^C-labeled methylglyoxal

[2-^13^C]MeGx was synthesized by adapting methods used previously for radiolabeling^20,31^. Namely, 2.5 g of selenium dioxide (Fluka, Germany) was dissolved in 10 g of MilliQ water (Merck Millipore, Burlington, MA, USA). Then, 1 g of [2-^13^C]acetone was added and the mixture was brought to 60 °C in an evacuated vial. Shortly after, a red precipitate began to form, and the reaction mixture was kept at 60˚C for 8 h. After cooling to room temperature, the yellow-orange liquid was distilled under reduced pressure. A ^1^H NMR spectrum of the distillate boiling at 56–60 °C revealed a mixture that consisted mostly of water and methylglyoxal, with small amounts of acetate and formate. Finally, the distillate was freeze-dried overnight, which removed most of the water, acetate and formate, leaving a pale-yellow viscous oil-like liquid. Samples were stored at −20 °C until use. After dissolving the final product in D_2_O, the ^1^H and ^13^C NMR spectra (Supplementary Figures 2-4) showed MeGx of high purity, with only small quantities of oligomeric species, which were decomposed into monomeric methylglyoxal upon incubation in H_2_O at room temperature^32^. The final-product yield was ~10%. The procedure for synthesis of [1,3-^13^C]MeGx was identical, with [1,3-^13^C]acetone used as a starting material.

### Preparation of cell cultures

Blood was obtained from consenting healthy donors, under Human Ethics Committee clearance. Heparin (15 U mL^−1^) was used as an anti-coagulant and blood was centrifuged at 3000 × *g* for 5 min at 4 °C. The plasma and buffy coat were removed by vacuum-pump aspiration. RBC pellets were washed three times in five volumes of phosphate-buffered saline (PBS) and prior to the last wash cell suspensions were bubbled with oxygen for 10 min, converting hemoglobin to diamagnetic oxyhemoglobin. Hematocrits were measured using a capillary centrifuge (12000 × *g*; Hermle, Z252M, Wehingen, Germany). Samples were stored at 4 °C for up to 24 h until used. In experiments with hemolysates, RBC suspensions were subjected to two cycles of freezing in liquid nitrogen and thawing at ~30 °C. Samples were typically used on the same day.

The murine lymphoma EL4 cells were cultured in Roswell Park Memorial Institute (RPMI) medium using the protocol provided by the supplier (ATCC, Manassas, VA, USA).

### NMR spectroscopy

Non-hyperpolarized ^1^H and ^13^C NMR spectra were recorded at 400.13 and 100.6 MHz, respectively, on a Bruker (Karlsruhe, Germany) Avance III console with a 9.4 T vertical wide-bore magnet from Oxford Instruments (Oxford, UK), equipped with a 10-mm Bruker TXI or a broadband (BBO) probe.

For the RD-DNP experiments with cell suspensions or lysates, a Bruker Avance II 600 MHz spectrometer (150.9 MHz for ^13^C) with a 14.1 T vertical magnet and 10-mm Bruker broadband probe were used to record the ^13^C NMR spectra. The ^13^C NMR RD-DNP spectra were acquired as reported recently;^23^ viz., each was based on a single free induction decay (FID) of 32,768 complex data points acquired with a repetition time of 1 s. FID acquisition time was 0.806 s; spectral width was 269.35 ppm; the carrier frequency was set to 120 ppm; the duration of the radio-frequency pulse was 1 μs, which corresponded to a nutation (flip) angle of 4^o^. The data were processed with one degree of zero filling and multiplication with an exponential line-broadening factor of 1 Hz before Fourier transformation. ^13^C NMR chemical shifts of each of MGBH and MGMH were assigned as in^33^. For the HTA and MG peaks, the assignment was done by adding extra GSH to a hemolysate, which increased one of the two peaks that was then assigned to HTA. NMR spectra were phased and baseline corrected in Bruker TopSpin 3.5, prior to being imported into *Mathematica*^34^ for post-processing.

### RD-DNP sample preparation

Approximately 30 mg (~0.4 mmol) of ^13^C-labeled MeGx was dissolved in 140 μL of 50:50 DMSO-d6 and D_2_O containing 15 mM trityl radical (tris(8-carboxy-2,2,6,6-tetra-(hydroxyethyl)-benzo-[1,2,4,5]-bis-(1,3)-dithiole-4-yl)-methyl sodium salt (OX063, GE Healthcare, Amersham, Buckinghamshire, UK) and 0.7 mM of gadolinium chelate (Dotarem; Guerbet Laboratories Ltd, Solihull, West Midlands, UK). The mixture was hyperpolarized using a 3.35 T HyperSense (Oxford Instruments, Abingdon, UK), at a temperature of ~1.2 K using microwaves with a frequency of 94.115 GHz. The sample underwent rapid dissolution in 6 mL of a solution containing 10 mM Na phosphate, 137 mM NaCl and 2 mM KCl (pH 7.4 at 37 °C, at 10 bar and 180˚C) to give a final MeGx concentration of ~80 mM. Up to 2 mL of the hyperpolarized solution was injected via a heat-exchange apparatus^23^ into samples of cells or lysates, which were pre-equilibrated at 37 °C in a 10-mm NMR tube. The time from sample ejection to injection into the lysate/cell-suspension was typically ~17 s. For the experiments on anaesthetized mice, after a similar delay, 100 μL of the hyperpolarized solution was injected via a caterer into the tail vein over ~10 s.

### Tumor implantation

Animal experiments were conducted in compliance with personal and project licenses issued under the UK Animals (Scientific Procedures) Act, 1986. Experimental and handling protocols were approved by the Cancer Research UK, Cambridge Institute Animal Welfare and Ethical Review Body.

As in^35^, tumors were initiated by subcutaneous injection of 5 × 10^6^ viable EL4 murine lymphoma cells in 100 μL in the left flank of C57BL/6 female mice (Charles River Ltd., Harlow, Essex, UK) at 6-8 weeks of age and weighing ~20 g. Experiments were conducted when the tumor volume was ~2 mL, typically 10 days after implantation. The animals were fed ad libitum on standard mouse chow. They were anaesthetized with inhalation of 1–2% isoflurane (IsoFlo, Abbott Laboratories Ltd., Maidenhead, Berkshire, UK) in air/O_2_ (75/25%, 2 L min^−1^). Body temperature was maintained with warm air blown though the magnet bore and was recorded with a rectal thermometer. Breathing rate (~80 cycles min^−1^) and temperature were recorded with a purpose-built device from Biotrig (Small Animal Instruments, Stony Brook, New York, USA).

### In vivo magnetic resonance spectroscopy

Experiments were conducted on a 7 T horizontal-bore magnet (Agilent, Palo Alto, CA, USA) using an actively decoupled double-tuned ^13^C-^1^H volume transmit coil (Rapid Biomedical, GmbH, Rimpar, Germany; inside diameter 42 mm) and a 20-mm diameter ^13^C surface receiver coil (Rapid Biomedical). Tumors were located with transverse ^1^H images acquired with a spin-echo pulse sequence using a repetition time of 1.5 s, echo time of 10 ms, a field of view of 40 × 40 mm, a data matrix of 128 × 128 complex points, a slice thickness of 2 mm, and 15 slices. Time courses were recorded as single transient spectra from a 6-mm thick slice of the tumor (or brain or liver) acquired using a slice-selective excitation pulse with an estimated flip-angle of 10^o^. Using a repetition time of 1 s, 180 spectra were acquired in 3 min. The data were imported into a MatLab (Mathworks, MA, USA) script and peaks were integrated. Further analyses including graphical output were conducted in *Mathematica*^34^.

### Quantitative analysis of time-course data

Initially, the glyoxalase pathway reactions were modeled using the scheme shown in Supplementary Figure 11a, which included ‘unlabeled’ (non-hyperpolarized) species. Each kinetic step was accounted for by a first-order rate constant, with *k*_Glo1_ and *k*_Glo2_ denoting equivalent apparent first-order rate constants that characterize the two enzymes. Since the peak intensities of the ketoaldehyde form (MG) and HTA were low, and attempts to include them in the fitting process failed, we switched to a reduced model (Supplementary Figure 11b). The extent of reaction was sufficiently small that initial velocities could be estimated, including those of the glyoxalase reactions, by nonlinear regression analysis using only the equations describing the time dependence of the four major hyperpolarized species (Fig. 2c). Attempts to incorporate the application of the small flip-angle readout pulse, α, into the model showed that the presence of the angle only affected the values of the apparent longitudinal relaxation times, *T*_1_, in a predictable way (Supplementary Tables 1-7), as described in^36^. Thus, for efficiency of the numerical procedures, the final fits were performed by omitting application of α.

The Michaelis–Menten parameters were initially estimated in terms of the total concentration of the injected methylglyoxal, [MeGx], (Fig. 3):

$$k_{{\mathrm{Glo}}1} = \frac{{V_{{\mathrm{max}}}^{{\mathrm{Glo}}1,{\mathrm{app}}}}}{{K_{\mathrm{m}}^{{\mathrm{Glo}}1,{\mathrm{app}}} + [{\mathrm{MeGx}}]}}$$

$$k_{{\mathrm{Glo}}2} = \frac{{V_{{\mathrm{max}}}^{{\mathrm{Glo}}2,{\mathrm{app}}}}}{{K_{\mathrm{m}}^{{\mathrm{Glo}}2,{\mathrm{app}}} + [{\mathrm{MeGx}}]}}$$

However, the true substrate of Glo1 is HTA, while the true substrate of Glo2 is SLG. Moreover, GSH is a competitive inhibitor of Glo1, while HTA is a competitive inhibitor of Glo2^15^. Thus, the expressions for the rates of the two enzymes are of the following Michaelis–Menten forms:^15^

$$\upsilon _{{\mathrm{Glo}}1} = \frac{{V_{{\mathrm{max}}}^{{\mathrm{Glo}}1}[{\mathrm{HTA}}]}}{{K_{\mathrm{m}}^{{\mathrm{Glo}}1}\left( {1 + \frac{{[{\mathrm{GSH}}]}}{{K_{\mathrm{i}}^{{\mathrm{GSH}}}}}} \right) + [{\mathrm{HTA}}]}}$$

$$\upsilon _{{\mathrm{Glo}}2} = \frac{{V_{{\mathrm{max}}}^{{\mathrm{Glo}}2}\left[ {{\mathrm{SLG}}} \right]}}{{K_{\mathrm{m}}^{{\mathrm{SLG}}}\left( {1 + \frac{{\left[ {{\mathrm{HTA}}} \right]}}{{K_{\mathrm{i}}^{{\mathrm{HTA}}}}}} \right) + \left[ {{\mathrm{SLG}}} \right]}}.$$

The true substrate concentrations can be expressed via the dissociation equilibrium constants *K*_1_, *K*_2_ and *K*_3_, as follows from Fig. 1:

$$\frac{{\left[ {{\mathrm{MGBH}}} \right]_{{\mathrm{eq}}}}}{{\left[ {{\mathrm{MGMH}}} \right]_{{\mathrm{eq}}}}} = \frac{{k_{ - 1}}}{{k_1}} = K_1;\,{\mathrm{hence}}\,\left[ {{\mathrm{MGBH}}} \right]_{{\mathrm{eq}}} = K_1[{\mathrm{MGMH}}]_{{\mathrm{eq}}}$$

$$\frac{{\left[ {{\mathrm{MGMH}}} \right]_{{\mathrm{eq}}}}}{{\left[ {{\mathrm{MG}}} \right]_{{\mathrm{eq}}}}} = \frac{{k_{ - 2}}}{{k_2}} = K_2;\,{\mathrm{hence}}\,\left[ {{\mathrm{MGMH}}} \right]_{{\mathrm{eq}}} = K_2[{\mathrm{MG}}]_{{\mathrm{eq}}}$$

$$\frac{{\left[ {{\mathrm{MG}}} \right]_{{\mathrm{eq}}}\left[ {{\mathrm{GSH}}} \right]_{{\mathrm{eq}}}}}{{\left[ {{\mathrm{HTA}}} \right]_{{\mathrm{eq}}}}} = \frac{{k_{ - 3}}}{{k_3}} = K_3;{\mathrm{hence}}\,\left[ {{\mathrm{MG}}} \right]_{{\mathrm{eq}}} = \frac{{K_3\left[ {{\mathrm{HTA}}} \right]_{{\mathrm{eq}}}}}{{\left[ {{\mathrm{GSH}}} \right]_{{\mathrm{eq}}}}}$$

$$[{\mathrm{MeGx}}] = \left[ {{\mathrm{MGBH}}} \right]_{{\mathrm{eq}}} + \left[ {{\mathrm{MGMH}}} \right]_{{\mathrm{eq}}} + \left[ {{\mathrm{MG}}} \right]_{{\mathrm{eq}}} = \left( {K_1 + 1} \right)K_2[{\mathrm{MG}}]_{{\mathrm{eq}}} + \left[ {{\mathrm{MG}}} \right]_{{\mathrm{eq}}}$$

$$[{\mathrm{MeGx}}] = \left[ {\left( {K_1 + 1} \right)K_2 + 1} \right]\left[ {{\mathrm{MG}}} \right]_{{\mathrm{eq}}} = \frac{{[\left( {K_1 + 1} \right)K_2 + 1]K_3\left[ {{\mathrm{HTA}}} \right]_{{\mathrm{eq}}}}}{{\left[ {{\mathrm{GSH}}} \right]_{{\mathrm{eq}}}}}$$

$$\left[ {{\mathrm{HTA}}} \right]_{{\mathrm{eq}}} = \frac{{[{\mathrm{MeGx}}]\left[ {{\mathrm{GSH}}} \right]_{{\mathrm{eq}}}}}{{[\left( {K_1 + 1} \right)K_2 + 1]K_3}},$$

where the subscript ‘eq’ denotes equilibrium. Assuming that a quasi-equilibrium pertains, we omit the ‘eq’ subscript and obtain the following expressions for the rate constants in terms of [MeGx]:

$$\begin{array}{l}k_{{\mathrm{Glo}}1} = \frac{{V_{{\mathrm{max}}}^{{\mathrm{Glo}}1}}}{{K_{\mathrm{m}}^{{\mathrm{Glo}}1}\left( {1 + \frac{{[{\mathrm{GSH}}]}}{{K_{\mathrm{i}}^{{\mathrm{GSH}}}}}} \right) + \frac{{[{\mathrm{MeGx}}]\left[ {{\mathrm{GSH}}} \right]}}{{[\left( {K_1 + 1} \right)K_2 + 1]K_3}}}} = \\ = \frac{{\left( {\frac{{V_{{\mathrm{max}}}^{{\mathrm{Glo}}1}[\left( {K_1 + 1} \right)K_2 + 1]K_3}}{{[{\mathrm{GSH}}]}}} \right)}}{{\frac{{K_{\mathrm{m}}^{{\mathrm{Glo1}}}[\left( {K_1 + 1} \right)K_2 + 1]K_3}}{{[{\mathrm{GSH}}]}}\left( {1 + \frac{{[{\mathrm{GSH}}]}}{{K_{\mathrm{i}}^{{\mathrm{GSH}}}}}} \right) + [{\mathrm{MeGx}}]}}\end{array}.$$

Thus, for estimating the Michaelis–Menten parameters for Glo1, the obtained apparent value $K_{\mathrm{m}}^{{\mathrm{Glo}}1,{\mathrm{app}}}$ corresponds to $\frac{{K_{\mathrm{m}}^{{\mathrm{Glo}}1}[\left( {K_1 + 1} \right)K_2 + 1]K_3}}{{[{\mathrm{GSH}}]}}\left( {1 + \frac{{[{\mathrm{GSH}}]}}{{K_{\mathrm{i}}^{{\mathrm{GSH}}}}}} \right)$, while $V_{{\mathrm{max}}}^{{\mathrm{Glo}}1,{\mathrm{app}}}$ corresponds to $\left( {\frac{{V_{{\mathrm{max}}}^{{\mathrm{Glo}}1}[\left( {K_1 + 1} \right)K_2 + 1]K_3}}{{[{\mathrm{GSH}}]}}} \right)$. These expressions were employed in the calculations, using previously determined values of *K*_1_, *K*_2_, *K*_3_, $K_{\mathrm{i}}^{{\mathrm{GSH}}}$ (0.412, 0.01925, 10.67 mM and 7.88 mM, respectively^15^) and [GSH] = ~2 mM in RBCs^25^.

### General computations

Progress curves of NMR spectral intensity were fitted by straight lines or exponential curves using the *Mathematica*^34^ function NonlinearModelFit. When comparing two different slopes for a statistically-significant difference the standard two-tailed *t*-test was used^37^.

## Supplementary information

Supplementary Information

## Data Availability

The data sets generated during and/or analyzed during the current study are available from the corresponding author on reasonable request.
