# Supplementary material for: Glyoxalase activity in human erythrocytes and mouse lymphoma, liver and brain probed with hyperpolarized 13C-methylglyoxal
Source: Commun Biol. 2018 Dec 21;1:232. doi: 10.1038/s42003-018-0241-1 (PMC6303249; doi:10.1038/s42003-018-0241-1)
Supplement: Supplementary file 1 — Supplementary Information [file 42003_2018_241_MOESM1_ESM.pdf]

Supplementary Information

Supplementary Figures

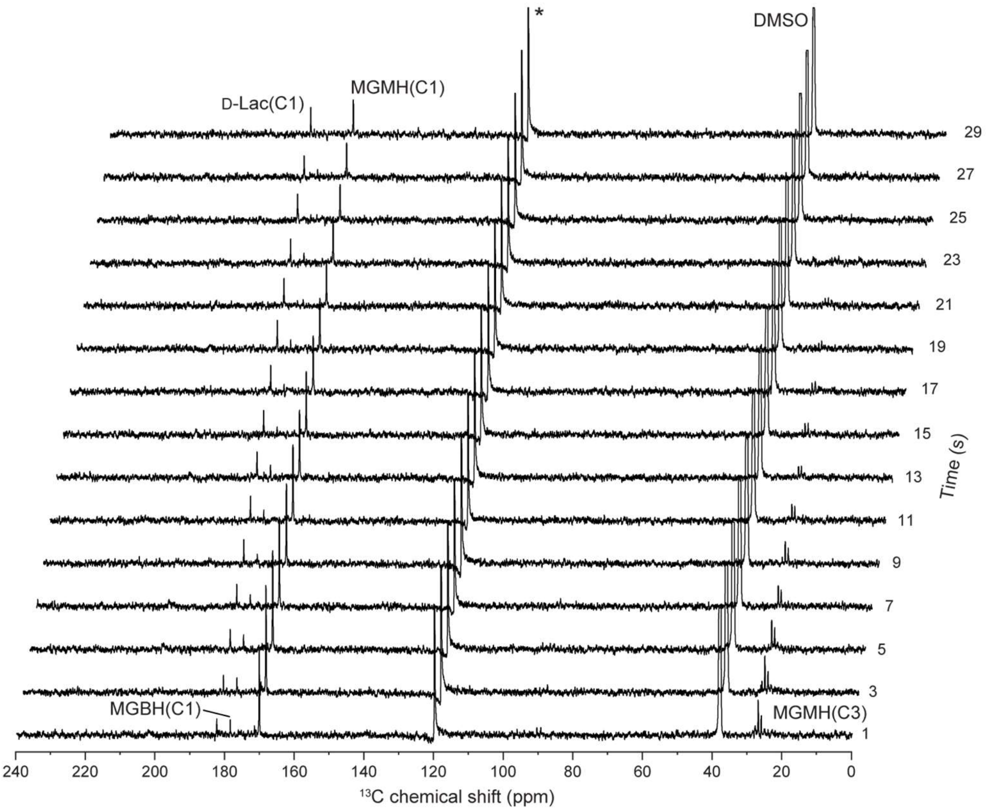

Supplementary Figure 1. RD-DNP time course of ^{13}C NMR spectra obtained upon injection of [1,3-^{13}C]methylglyoxal into a suspension of RBCs at 37°C. Two mL of hyperpolarized [1,3-^{13}C]MeGx of final concentration 23.6 mM was injected into 2 mL of RBCs (final *Ht* = 39%) in phosphate buffered saline. Spectra were acquired every 1 s. *Denotes the position of a 'central-frequency' artifact. *Abbreviations*: D-Lac(C1), C1 resonance of [1,3-^{13}C]D-lactate; MGBH(C1), C1 resonance of the bishydrate of [1,3-^{13}C]MeGx; MGMH(C1), C1 resonance of the monohydrate of [1,3-^{13}C]MeGx; MGMH(C3), C3 resonance of monohydrate of [1,3-^{13}C]MeGx; DMSO, natural abundance $^{13}\underline{C}D_3$ in the dimethylsulfoxide-d6 that was used to make up the hyperpolarization solution;. For clarity, the intense DMSO peak was truncated.

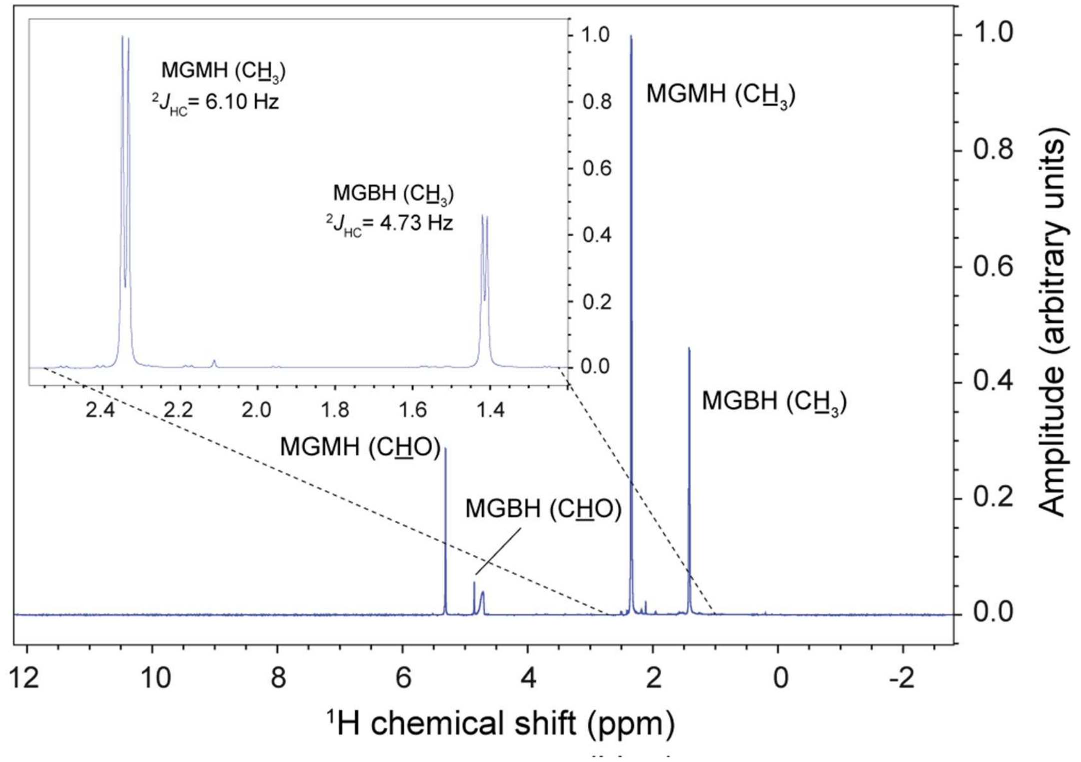

Supplementary Figure 2. ^{1}H NMR spectrum of [2-^{13}C]methylglyoxal in ~95% D_2O at 37˚C. Spectrum was acquired using a 90˚ excitation pulse and pre-saturation of the HOD resonance at 4.703 ppm during the relaxation delay. 32,768 complex points were recorded, and 64 transients per FID were collected with an inter-transient delay of 10 s, leading to a total experiment time of 13.5 min. The FID duration was 2.73 s. A line broadening factor of 1 Hz was applied before Fourier transformation, and the spectrum was zero-filled twice. The four major peaks in the spectrum were: 5.31 ppm (s, 1H, acetal H, MGMH), 4.85 ppm (s, 1H, acetal H, MGBH), 2.34 ppm (d, 6.10 Hz, 3H, CH_3, MGMH), 1.41 ppm (d, 4.73 Hz, 3H, CH_3, MGBH). The inset shows the zoomed doublets at 2.34 ppm and 1.41 ppm from the methyl hydrogen-nuclei of MGMH and MGBH, respectively.

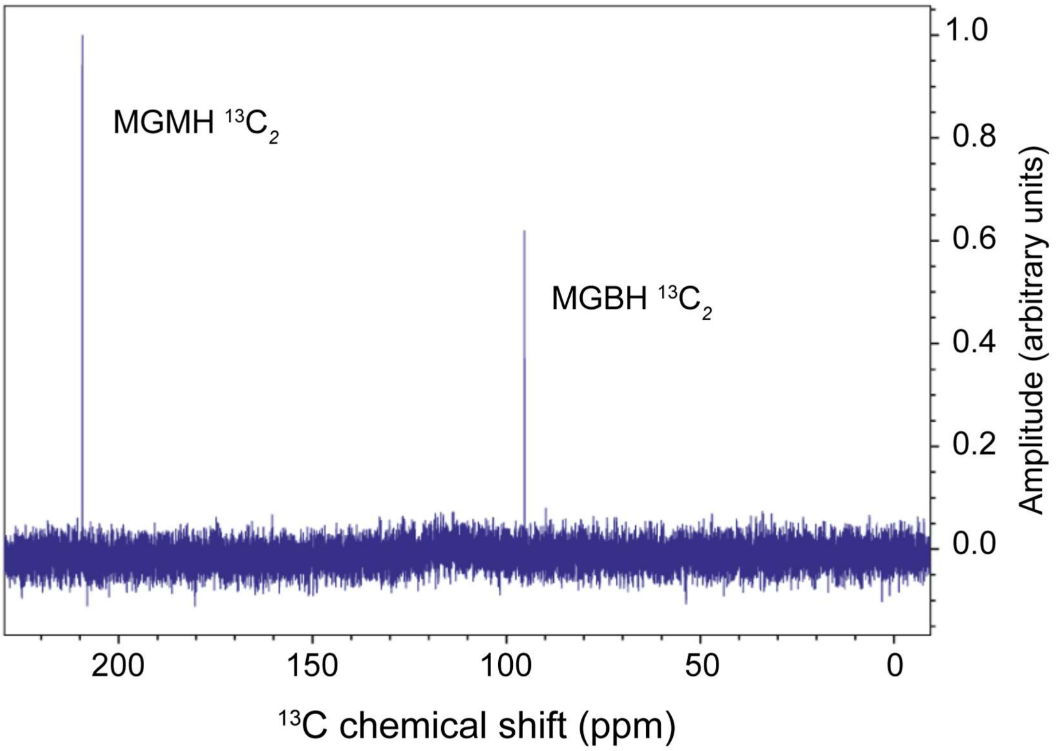

Supplementary Figure 3. ^{13}C NMR spectrum of ~40 mM [2-^{13}C]methylglyoxal in ~95% D_2O at 37°C. The spectrum was recorded with an excitation flip angle of 45°, using 65536 complex points with 4 dummy transients, 64 transients per FID and an inter-transient delay of 2 s, leading to a total experimental time of 4 min. The FID duration time was 1.36 s. ^{1}H decoupling was applied during the recording of the FID and relaxation delay. Line broadening of 1 Hz was applied before Fourier transformation and the spectrum was zero-filled once. Note the spectrum contains only the singlets from MGMH and MGBH thus attesting to the purity (at the level allowed by the signal-to-noise ratio) of the methylglyoxal preparation.

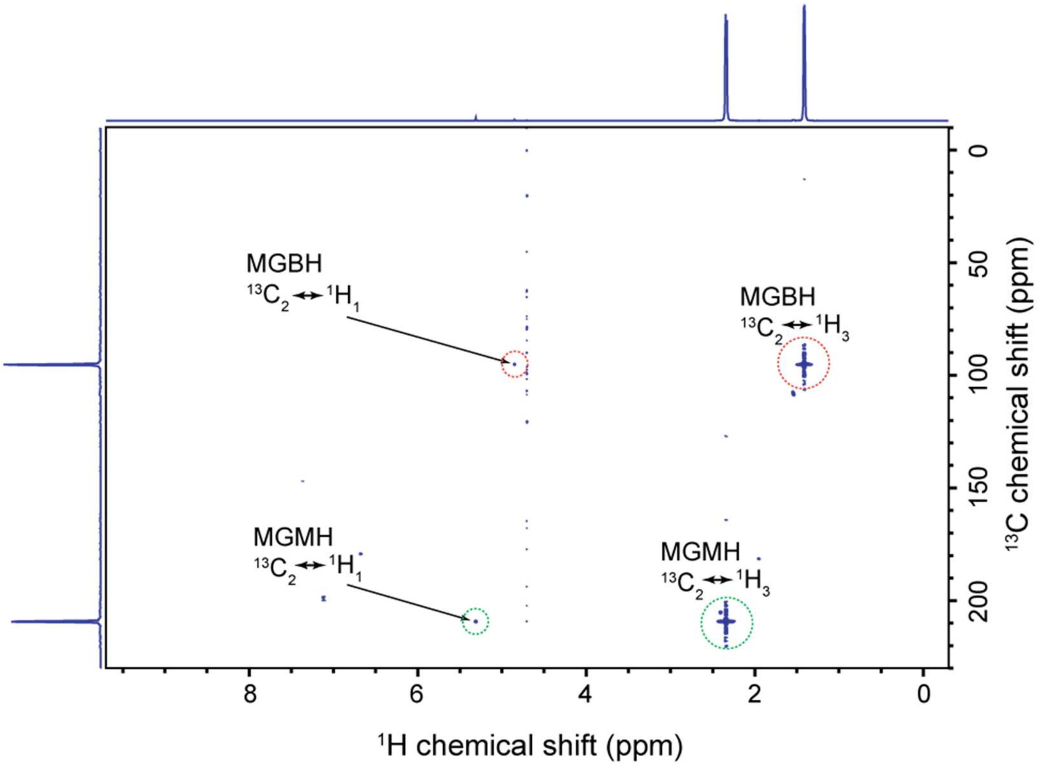

Supplementary Figure 4. ^{1}H-^{13}C heteronuclear multiple bond correlation (HMBC) spectrum of [2-^{13}C]methylglyoxal in ~95% D_2O at 37˚C. The spectrum was acquired using a data matrix of 2048 × 256 complex points, with 16 dummy transients, 4 transients per FID, and an inter-transient delay of 1 s, leading to a total experimental time of 55 min. The acquisition time in the direct and indirect dimension was 512.0 and 10.6 ms, respectively. The spectrum was processed in 'magnitude mode', and linear prediction was used in the indirect dimension to produce a matrix of 2048 × 1024 real data points. Dashed circles indicate cross peaks corresponding to the long-range ^{1}H-^{13}C correlations in MGMH and MGBH.

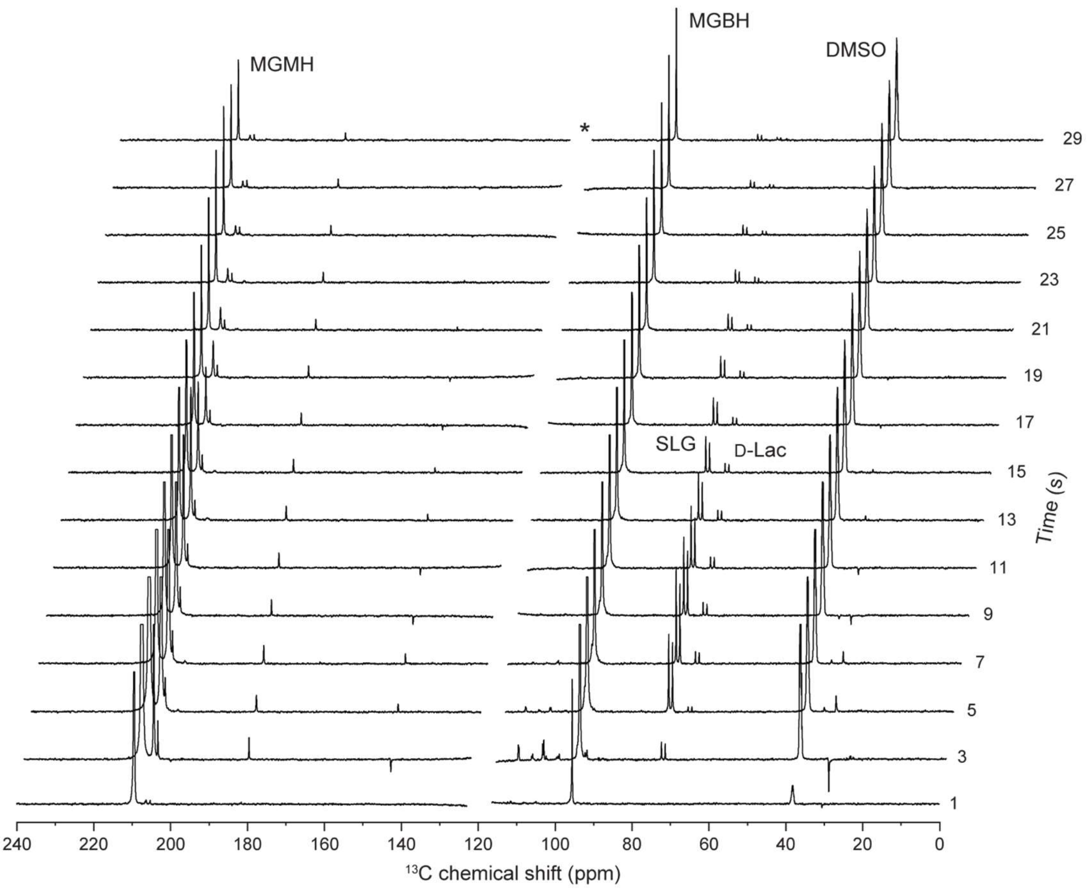

Supplementary Figure 5. RD-DNP time course of ^{13}C NMR spectra obtained upon injecting [2-^{13}C]methylglyoxal into a lysate of RBCs at 37°C. Two mL of hyperpolarized methylglyoxal of final concentration 17.7 mM was injected into 2 mL of hemolysate (final *Ht* = 39.5%) in phosphate buffered saline with addition of 5 mM reduced glutathione. Spectra were acquired every 1 s. *Denotes the position of a 'central-frequency' artifact that was edited out. *Abbreviations*: D-Lac, [2-^{13}C]D-lactate; MGBH, the bishydrate of [2-^{13}C]MeGx; MGMH, the monohydrate of [2-^{13}C]MeGx; SLG, [2-^{13}C]S-D-lactoylglutathione; DMSO, natural abundance $^{13}\underline{C}D_3$ in the dimethylsulfoxide-d6 that was used to make up the hyperpolarization solution. For clarity, the intense peaks of MGBH, MGMH and DMSO were truncated in the few initial spectra.

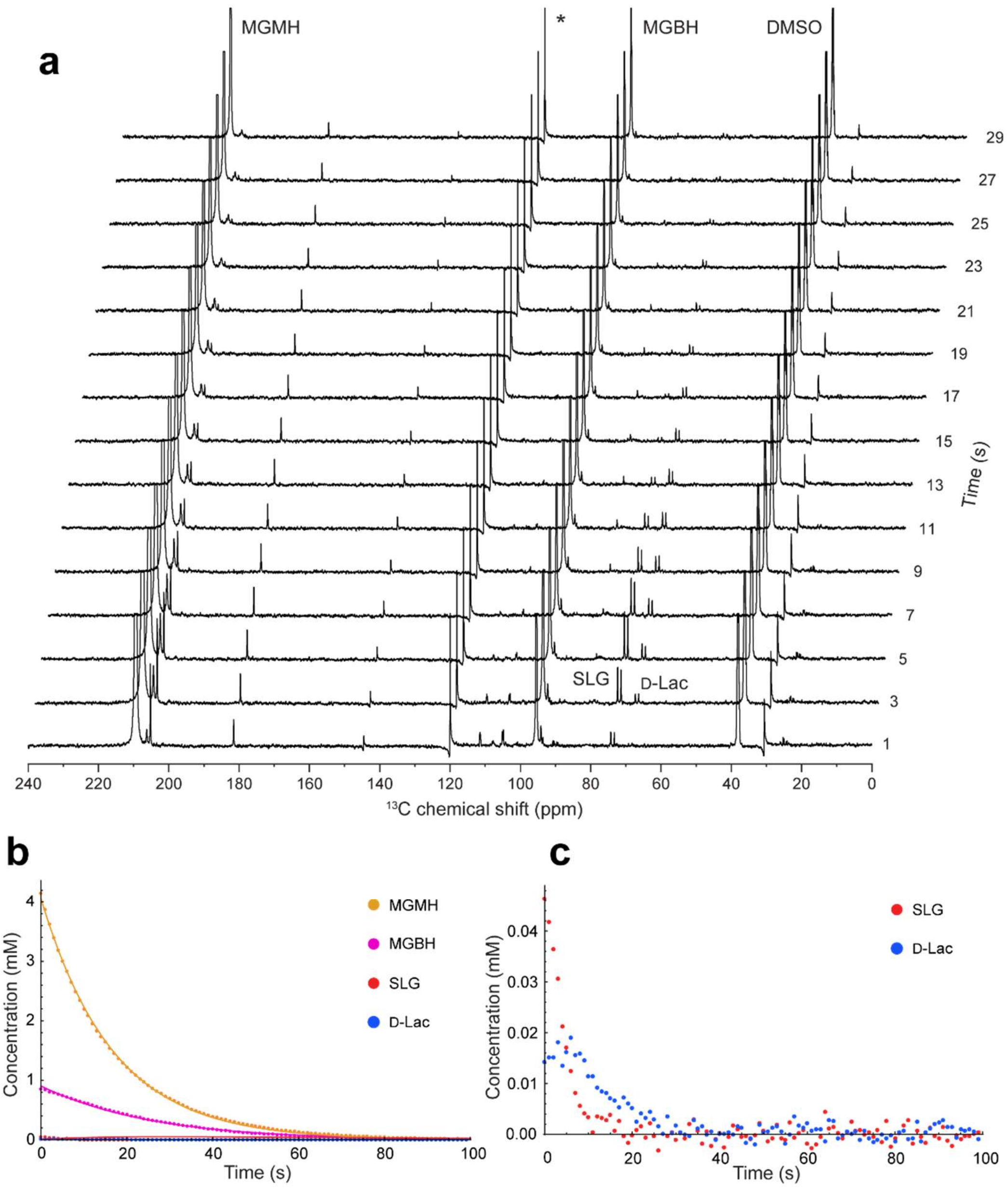

Supplementary Figure 6. RD-DNP time course obtained upon injecting [2-^{13}C]methylglyoxal into a suspension of murine lymphoma EL4 cells at 37°C. **a** A series of ^{13}C NMR spectra acquired upon injecting 2 mL of hyperpolarized [2-^{13}C]MeGx of final concentration 5.0 mM into 2 mL of EL4 cells of final cytocrit 9.25% in RPMI medium. Spectra were acquired every 1 s. *Denotes the position of a 'central-frequency' artifact. For clarity, the MGBH, MGMH and DMSO peaks were clipped. **b** Time course of ^{13}C NMR peak intensities from the spectra of **a**, converted to concentration values. **c** 100-fold zoom of the concentration scale highlighting the time courses of SLG and D-Lac shown in **b**. *Abbreviations*: D-Lac, [2-^{13}C]D-lactate; MGBH, the bishydrate of [2-^{13}C]MeGx; MGMH, the monohydrate of [2-^{13}C]MeGx; SLG, [2-^{13}C]S-D-lactoylglutathione; DMSO, natural abundance $^{13}\underline{C}D_3$ in the dimethylsulfoxide-d6 that was used to make up the hyperpolarization solution.

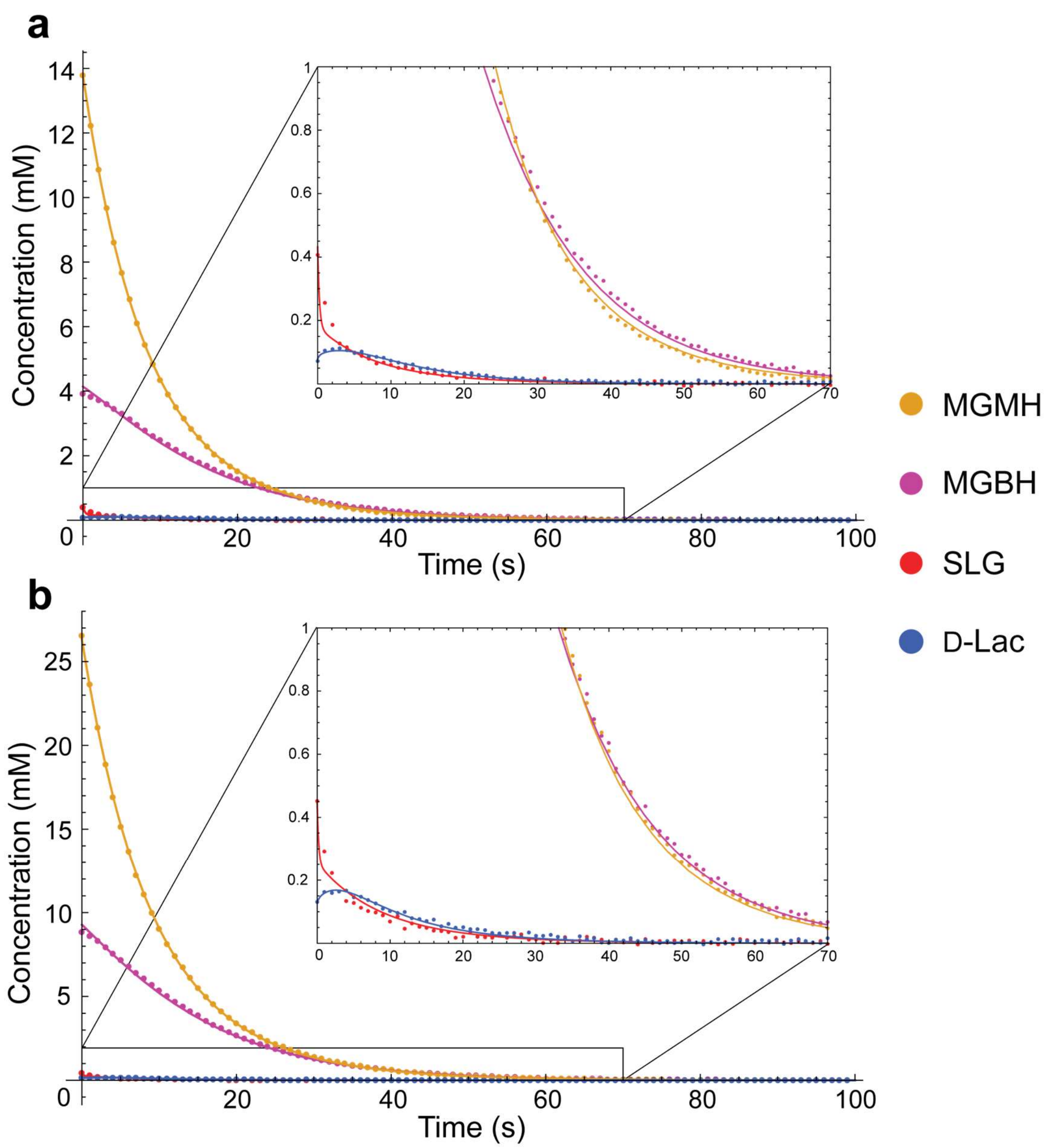

Supplementary Figure 7. RD-DNP time courses acquired following injection of hyperpolarized [2-^{13}C]methylglyoxal into a suspension of RBCs (final *Ht* = 39.5%). **a**, 17.7 mM [2-^{13}C]MeGx and **b**, 35.4 mM [2-^{13}C]MeGx. The other details of the experiment are the same as for Fig. 2.

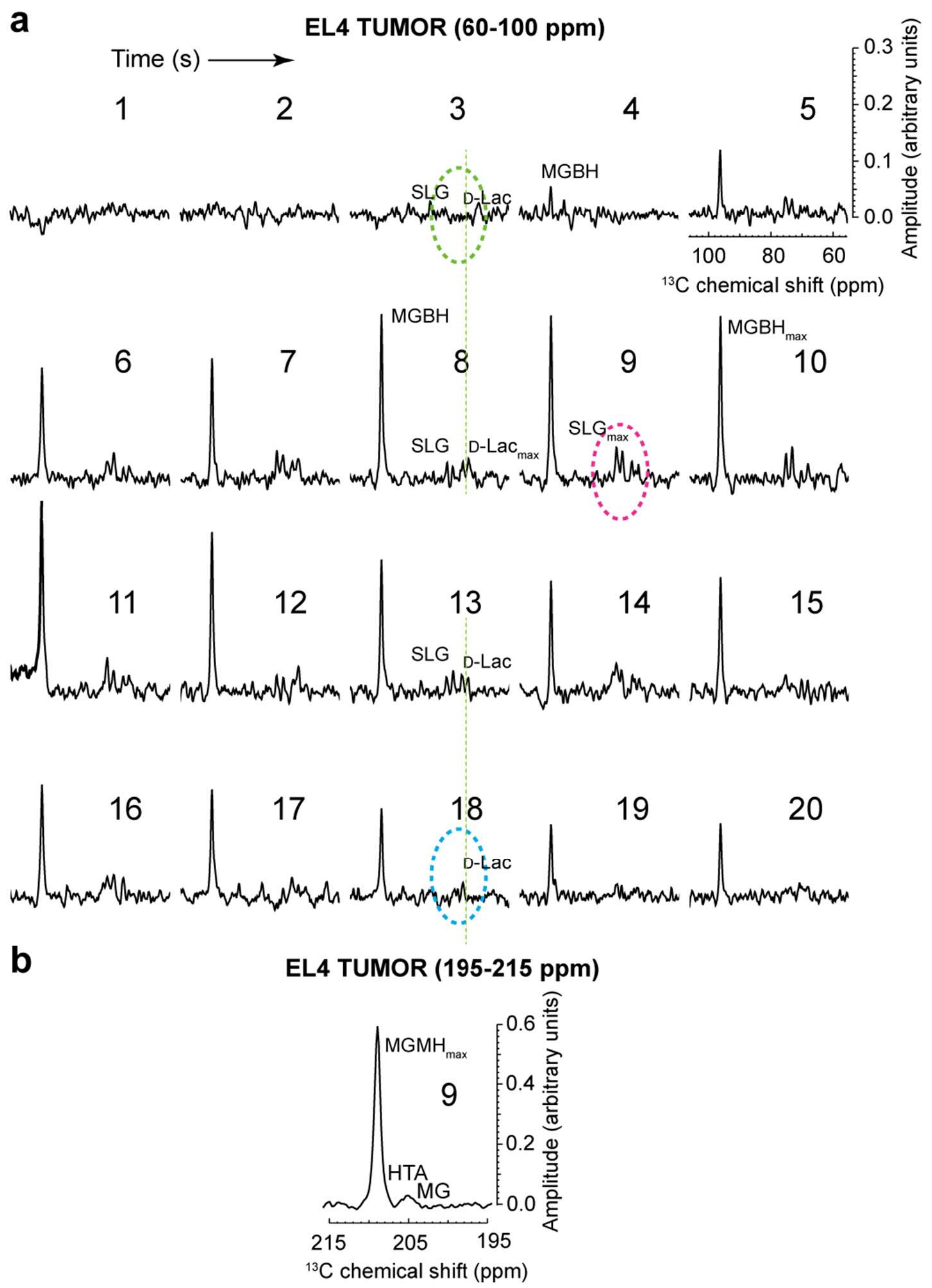

Supplementary Figure 8. In vivo ^{13}C NMR RD-DNP spectra at 75.4 MHz (7 T) acquired from an EL4 tumor in the flank of a live mouse that recovered from the experiment. (Qualitatively and quantitatively similar spectra were obtained from implanted EL4 tumors in two other mice.) Hyperpolarized [2-^{13}C]MeGx was injected into the tail vein in a volume of 100 μL. The spectra were acquired every 1 s for a time course of 180 s from the anaesthetized mouse. **a** Twenty spectra spanning 60-100 ppm, encompassing the domain of D-Lac, SLG, and MGBH. **b** A spectrum spanning 195-215 ppm, encompassing the domain of MG, HTA, and MGMH, with maximal MGMH intensity ($MGMH_{max}$) occurring 9 s after the injection of the hyperpolarized [2-^{13}C]MeGx.

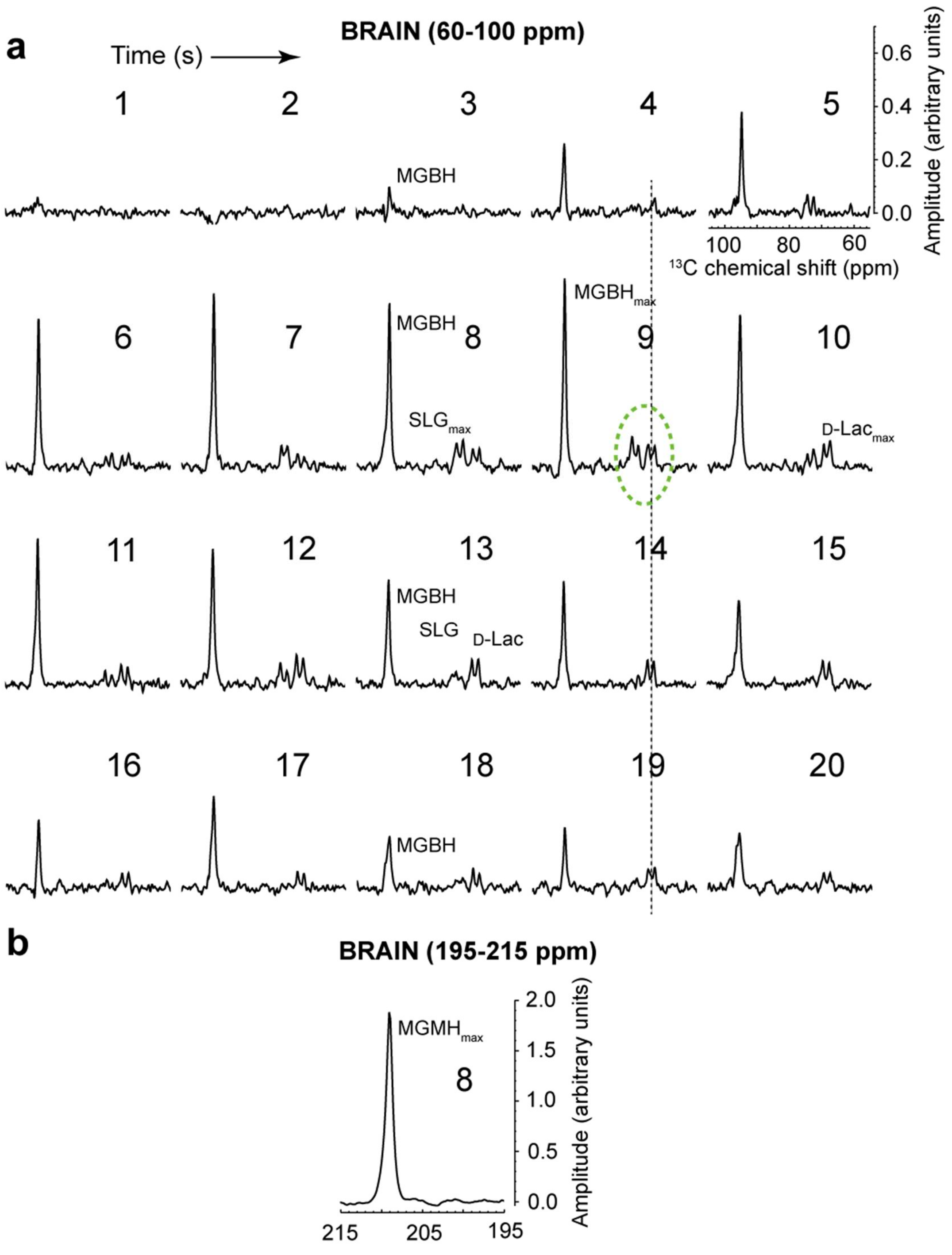

Supplementary Figure 9. In vivo ^{13}C NMR RD-DNP spectra at 75.4 MHz (7 T) acquired from the brain of a live mouse that recovered from the experiment. Hyperpolarized [2-^{13}C]MeGx was injected into the tail vein in a volume of 100 μL. The spectra were acquired every 1 s for a time course of 180 s from the anaesthetized mouse. **a** Twenty spectra spanning 60-100 ppm, encompassing the domain of D-Lac, SLG, and MGBH. **b** A spectrum spanning 195-215 ppm, encompassing the domain of MG, HTA, and MGMH, with maximal MGMH intensity ($MGMH_{max}$) occurring 8 s after the injection of the hyperpolarized [2-^{13}C]MeGx.

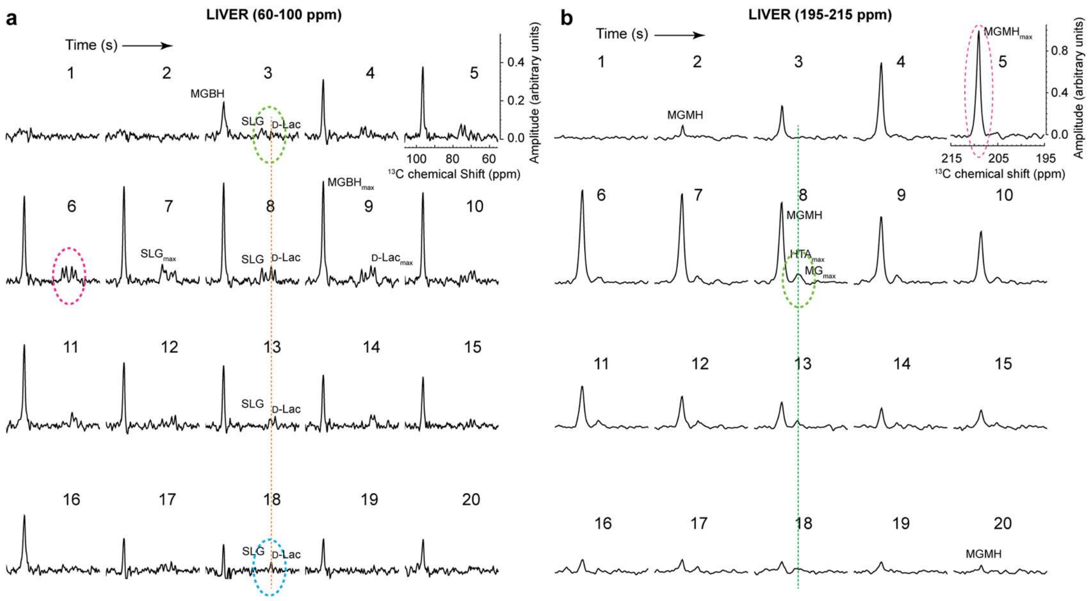

Supplementary Figure 10. In vivo ^{13}C NMR RD-DNP spectra at 75.4 MHz (7 T) acquired from the liver of a live mouse, which recovered from the experiment. **a** Spectra spanning 60-100 ppm, encompassing the domain of D-Lac, SLG, and MGBH. **b** Spectra spanning 195-215 ppm, encompassing the domain of MG, HTA, and MGMH. Hyperpolarized [2-^{13}C]MeGx was injected into the tail vein in a volume of 100 μL. The spectra were acquired every 1 s for a time course of 180 s from the anaesthetized mouse. A single spectrum from **a**, acquired at 6 s when the summed intensities of HTA and D-Lac was maximal, is given in Fig. 4. $MGBH_{max}$ indicates the spectrum that had the maximal intensity of MGBH, which occurred at 5 s after the injection.

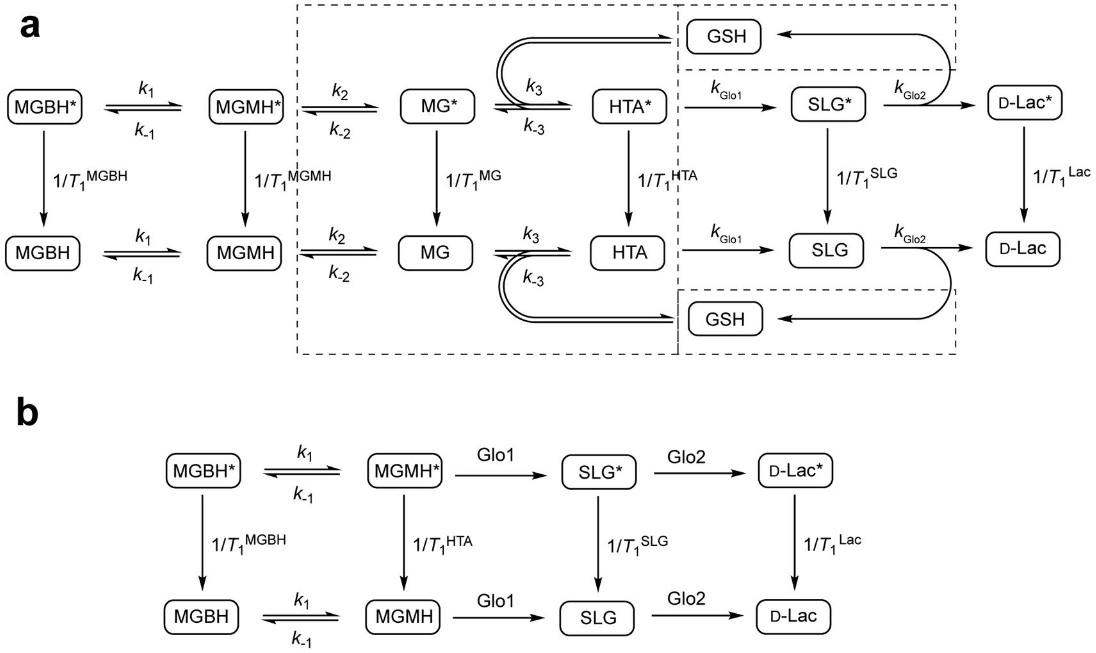

Supplementary Figure 11. Kinetic and nuclear magnetic relaxation pathways in ^{13}C-NMR RD-DNP experiments on [2-^{13}C]methylglyoxal in hemolysates, and suspensions of RBCs and EL4 cells. **a** The upper row of six reactants (superscript * denotes the hyperpolarized form) are all observed in the spectra (*e.g.*, Fig. 2a), but GSH, which is an essential (recycled) endogenous reactant is not, as implied by not having * on its label. The three dashed boxes surround reactants whose signals were not able to be quantified with sufficient precision to be used in kinetic analyses of the overall production rate of D-lactate. The rate constants characterizing the biochemical reactions are denoted by k_i i = ±1,…,±3, the rate of the glyoxalase-catalyzed reactions by k_{Glo1} and k_{Glo2}, and the NMR relaxation rate constants are the reciprocals of the corresponding longitudinal relaxation times (T_1) with a post-superscript to identify the particular molecular species. **b** Because the ketoaldehyde form (MG) and HTA were not able to be reliably quantified, and they represented a small fraction of the total ^{13}C-label pool, the kinetic system was simplified to this form with only four reactants used in all subsequent data analysis.

Supplementary Tables

Supplementary Table 1. Best-fit parameter values, obtained by fitting the model that incorporated a small flip-angle readout pulse, α. The value of α was changed from 0 to 10˚ in 2˚ increments. The RD-DNP dataset was obtained after injecting [2-^{13}C]MeGx to a final concentration of 5.0 mM into a suspension of EL4 cells (cytocrit 9.25%) in RPMI medium.

α, °	T_1^{MGBH}, s	T_1^{MGMH}, s	k_{-1}, s^{-1}	k_1, s^{-1}	[MGBH*](0), mM	[MGMH*](0), mM
0	34.8	15.9	0.009	0.039	0.834	3.867
2	35.6	16.0	0.009	0.039	0.834	3.867
4	38.1	16.5	0.009	0.039	0.834	3.867
6	43.1	17.4	0.009	0.039	0.834	3.867
8	52.9	18.8	0.009	0.039	0.834	3.867
10	74.6	21.0	0.009	0.039	0.834	3.867

Supplementary Table 2. Best-fit parameter values, obtained by fitting the model that incorporated a small flip-angle readout pulse, α. The value of α was changed from 0 to 10˚ in 2˚ increments. The RD-DNP dataset was obtained after injecting [2-^{13}C]MeGx to a final concentration of 5.0 mM into a suspension of RBCs (*Ht* 9.25%) in RPMI medium.

α, °	T_1^{MGBH}, s	T_1^{MGMH}, s	k_{-1}, s^{-1}	k_1, s^{-1}	[MGBH*](0), mM	[MGMH*](0), mM
0	20.5	14.5	0.020	0.043	0.874	3.790
2	20.7	14.6	0.020	0.043	0.874	3.790
4	21.6	15.1	0.020	0.043	0.874	3.790
6	23.1	15.8	0.020	0.043	0.874	3.790
8	25.6	16.9	0.020	0.043	0.874	3.790
10	29.8	18.7	0.020	0.043	0.874	3.790

Supplementary Table 3. Best-fit parameter values, obtained by fitting the model that incorporated a small flip-angle readout pulse, α. The value of α was changed from 0 to 10˚ in 2˚ increments. The RD-DNP dataset was obtained after injecting [2-^{13}C]MeGx to a final concentration of 5.0 mM into a suspension of RBCs (*Ht* 9.25%) in PBS medium.

α, °	T_1^{MGBH}, s	T_1^{MGMH}, s	k_{-1}, s^{-1}	k_1, s^{-1}	[MGBH*](0), mM	[MGMH*](0), mM
0	13.7	16.1	0.019	0.018	0.960	3.815
2	13.8	16.3	0.019	0.018	0.960	3.815
4	14.2	16.8	0.019	0.018	0.960	3.815
6	14.8	17.7	0.019	0.018	0.960	3.815
8	15.8	19.1	0.019	0.018	0.960	3.815
10	17.3	21.4	0.019	0.018	0.960	3.815

Supplementary Table 4. Best-fit parameter values, obtained by fitting the model that incorporated a small flip-angle readout pulse, α. The value of α was changed from 0 to 10˚ in 2˚ increments. The RD-DNP dataset was obtained after injecting [2-^{13}C]MeGx to a final concentration of 17.4 mM into a suspension of EL4 cells (cytocrit 12.5%) in RPMI medium.

α, °	T_1^{MGBH}, s	T_1^{MGMH}, s	k_{-1}, s^{-1}	k_1, s^{-1}	[MGBH*](0), mM	[MGMH*](0), mM
0	20.9	17.3	0.012	0.030	3.877	12.484
2	21.1	17.5	0.012	0.030	3.877	12.484
4	22.0	18.1	0.012	0.030	3.877	12.484
6	23.6	19.2	0.012	0.030	3.877	12.484
8	26.3	20.9	0.012	0.030	3.877	12.484
10	30.7	23.6	0.012	0.030	3.877	12.484

Supplementary Table 5. Best-fit parameter values, obtained by fitting the model that incorporated a small flip-angle readout pulse, α. The value of α was changed from 0 to 10˚ in 2˚ increments. The RD-DNP dataset was obtained after injecting [2-^{13}C]MeGx to a final concentration of 17.7 mM into a suspension of RBCs (*Ht* 12.5%) in PBS medium.

α, °	T_1^{MGBH}, s	T_1^{MGMH}, s	k_{-1}, s^{-1}	k_1, s^{-1}	[MGBH*](0), mM	[MGMH*](0), mM
0	17.7	15.1	0.018	0.034	2.779	13.797
2	17.9	15.3	0.018	0.034	2.779	13.797
4	18.5	15.7	0.018	0.034	2.779	13.797
6	19.6	16.5	0.018	0.034	2.779	13.797
8	21.4	17.7	0.018	0.034	2.779	13.797
10	24.2	19.7	0.018	0.034	2.779	13.797

Supplementary Table 6. Best-fit parameter values, obtained by fitting the model that incorporated a small flip-angle readout pulse, α. The value of α was changed from 0 to 10˚ in 2˚ increments. The RD-DNP dataset was obtained after injecting [2-^{13}C]MeGx to a final concentration of 18.25 mM into a suspension of EL4 cells (cytocrit 3.5%) in RPMI medium.

α, °	T_1^{MGBH}, s	T_1^{MGMH}, s	k_{-1}, s^{-1}	k_1, s^{-1}	[MGBH*](0), mM	[MGMH*](0), mM
0	28.1	19.1	0.013	0.042	3.329	14.009
2	28.6	19.4	0.013	0.042	3.329	14.009
4	30.2	20.1	0.013	0.042	3.329	14.009
6	33.3	21.4	0.013	0.042	3.329	14.009
8	38.8	23.5	0.013	0.042	3.329	14.009
10	49.5	27.1	0.013	0.042	3.329	14.009

Supplementary Table 7. Best-fit parameter values, obtained by fitting the model that incorporated a small flip-angle readout pulse, α. The value of α was changed from 0 to 10˚ in 2˚ increments. The RD-DNP dataset was obtained after injecting [2-^{13}C]MeGx to a final concentration of 18.8 mM into a suspension of RBCs (Ht 3.5%) in PBS medium.

α, °	T_1^{MGBH}, s	T_1^{MGMH}, s	k_{-1}, s^{-1}	k_1, s^{-1}	[MGBH*](0), mM	[MGMH*](0), mM
0	35.8	17.0	0.019	0.061	3.639	14.239
2	36.6	17.2	0.019	0.061	3.639	14.239
4	39.2	17.7	0.019	0.061	3.639	14.239
6	44.5	18.8	0.019	0.061	3.639	14.239
8	55.0	20.4	0.019	0.061	3.639	14.239
10	79.0	23.0	0.019	0.061	3.639	14.239

Supplementary Table 8. Best-fit parameter values (mean ± SE), obtained from low cytocrit samples (used for estimation of T_1^{MGBH}, T_1^{MGMH}, k_{-1} and k_1 values) by fitting the model without incorporation of a small flip-angle readout pulse, α.

[MeGx](0), mM	Cell type/medium	Cytocrit, %	T_1^{MGBH}, s	T_1^{MGMH}, s	k_{-1}, s^{-1}	k_1, s^{-1}	[MGBH*](0), mM	[MGMH*](0), mM
5.0	EL4/RPMI	9.25	34.8 ± 1.3	15.9 ± 0.1	0.009 ± 0.0003	0.039 ± 0.001	0.855 ± 0.003	4.121 ± 0.003
5.0	RBC/RPMI	9.25	20.5 ± 0.4	14.5 ± 0.1	0.020 ± 0.0003	0.043 ± 0.001	0.874 ± 0.002	4.104 ± 0.002
5.0	RBC/PBS	9.25	13.7 ± 0.6	16.1 ± 0.3	0.019 ± 0.0010	0.018 ± 0.002	0.974 ± 0.007	4.118 ± 0.007
17.4	EL4/RPMI	12.5	20.9 ± 0.7	17.3 ± 0.2	0.012 ± 0.0005	0.030 ± 0.002	4.026 ± 0.008	13.266 ± 0.008
17.7	RBC/PBS	12.5	17.7 ± 0.2	15.1 ± 0.1	0.018 ± 0.0002	0.034 ± 0.001	2.777 ± 0.005	14.904 ± 0.005
18.25	EL4/RPMI	3.5	28.1 ± 0.5	19.1 ± 0.1	0.013 ± 0.0002	0.042 ± 0.001	3.397 ± 0.004	14.813 ± 0.004
18.8	RBC/PBS	3.5	35.8 ± 1.7	17.0 ± 0.2	0.019 ± 0.0004	0.061 ± 0.002	3.692 ± 0.010	15.152 ± 0.010

Supplementary Table 9. Best-fit parameter values (mean ± SE), were obtained from high cytocrit samples performed using fixed values of T_1^{MGBH} = 19.3 s, T_1^{MGMH} = 16.0 s, k_{-1} = 0.015 s^{-1} and k_1 = 0.039 s^{-1}, and fitting the model without incorporation of a small flip-angle readout pulse, α. All datasets were obtained by injecting hyperpolarized MeGx into suspensions of RBCs in PBS medium.

[MeGx](0), mM	*Ht*, %	T_1^{SLG}, s	T_1^{Lac}, s	k_{GLO1}, s^{-1}	k_{GLO2}, s^{-1}	[MGBH*](0), mM	[MGMH*](0), mM	[SLG*](0), mM	[Lac*](0), mM
1.05	39	1.14 ± 0.04	4.5 ± 0.1	0.313 ± 0.003	0.428 ± 0.019	0.292 ± 0.002	0.765 ± 0.004	0.194 ± 0.002	0.089 ± 0.004
2.1	39	1.04 ± 0.01	6.1 ± 0.2	0.250 ± 0.001	0.163 ± 0.008	0.687 ± 0.002	1.421 ± 0.001	0.038 ± 0.002	0.013 ± 0.004
4.7	40	0.88 ± 0.01	6.7 ± 0.4	0.175 ± 0.001	0.138 ± 0.011	1.289 ± 0.005	3.501 ± 0.008	0.601 ± 0.004	0.017 ± 0.010
9.4	40	0.57 ± 0.01	6.7 ± 0.3	0.086 ± 0.001	0.135 ± 0.001	2.242 ± 0.008	7.281 ± 0.007	0.531 ± 0.003	0.110 ± 0.012
17.7	39.5	0.24 ± 0.01	4.8 ± 0.2	0.054 ± 0.001	0.172 ± 0.003	4.140 ± 0.005	13.794 ± 0.005	0.432 ± 0.009	0.079 ± 0.006
35.4	39.5	0.21 ± 0.01	3.1 ± 0.3	0.047 ± 0.001	0.285 ± 0.004	9.279 ± 0.015	26.344 ± 0.015	0.449 ± 0.005	0.127 ± 0.009
